# Community pharmacists preparedness to control OTC medication abuse in Saudi Arabia: a nationwide cross-sectional survey-based study

**DOI:** 10.3389/fpubh.2025.1723181

**Published:** 2026-01-08

**Authors:** Mona Yaser Alsheikh, Ahmed Ibrahim Fathelrahman

**Affiliations:** 1 Pharmacy Practice Department, Faculty of Pharmacy, King Abdulaziz University, Jeddah, Saudi Arabia; 2 Clinical Pharmacy Department, College of Pharmacy, Taif University, Taif, Saudi Arabia

**Keywords:** abuse, BTC medications, community pharmacy, OTC medications, Saudi Arabia, vigilance

## Abstract

**Background:**

Over-the-counter (OTC) medication misuse, abuse, and addiction are increasingly recognized issues not only in Saudi Arabia but also worldwide. Investigating OTC medication abuse and pharmacists' preparedness to control it is an urgent research area that will help to establish strategies to reduce associated harms.

**Objective:**

To explore community pharmacists' awareness, views, and preparedness to control OTC medication's abuse in Saudi Arabia.

**Methods:**

This cross-sectional study was conducted between November 2021 and February 2022. Data were collected using an online self-administered questionnaire distributed to community pharmacists across Saudi Arabia.

**Results:**

A total of 813 out of 919 community pharmacists completed the survey (response rate: 88.5%). Most of them were male (702, 86.3%), non-Saudi (623, 76.6%), bachelor's degree holders (648, 79.7%), professionally classified as pharmacists (534, 65.7%), aged ≤ 40 years (756, 93.0%), and from the western region (363, 44.6%). Using a 5-point Likert scale, pharmacists perceived their knowledge of OTC medication abuse to be high (mean score 4.3±/0.9, 86.0%). OTC medication classes most perceived as carrying a risk of addiction were codeine-containing products (82.2%), followed by cough suppressants/antitussives (69.5%). Dealing with a suspicious patient/customer who may be abusing OTC medication, most respondents said they would provide advice to patients as necessary (707, 87.0%) and refer to a physician (696, 85.6%). The top-rated barriers to preventing OTC medication abuse were lack of patient records on OTC medication use (mean score 3.2±/1.4, 64.0%) and workload (mean score 3.1 ± 1.5, 62.0%).

**Conclusion:**

Most community pharmacists in Saudi Arabia are aware of OTC medication abuse. Establishment of clear policies and guidelines regarding purchasing limits and monitoring practices is urgently needed.

## 1 Introduction

Engagement in substance misuse, abuse, and addiction poses a serious threat to community health worldwide and places an additional burden on healthcare services (1–3). Over-the-counter (OTC) medications are drugs that can be acquired legally without a prescription to assist patients/consumers in self-management of minor illnesses and symptom relief. This makes OTC medications highly accessible and widely used; however, despite their relative safety compared with prescribed medications, OTC medications are not completely safe (4–6). From a pharmacoepidemiologic point-of-view, any medication can pose risks for certain people, in certain amounts, in certain situations. Some OTC medications are susceptible to misuse or abuse, particularly among adolescents and young adults, and greater proportions of medications abused can be addictive (1–3, 5). For instance, codeine and dextromethorphan- containing compounds, when used irrationally, lead to dependence, tolerance, and withdrawal symptoms (2, 5). Furthermore, some non-addictive medications can be physically or chemically modified into addictive substances using traditional or newly emerged techniques. Examples include ephedrine and diphenhydramine, which can be mixed to produce amphetamine-like compounds similar in effect to fenethylline (Captagon^®^) in action (7). The whole world, led by World Health Organization (WHO) and drug regulators such as Food and Drug and Drug Administration (FDA), is engaged in the fight to combat abuse and addiction (7, 8). In the Islamic countries, such behaviors are strongly condemned according to Islamic teachings and values that criticize and condemn self-harm and harm to others.

OTC medication misuse, abuse, and addiction are increasingly recognized as public health issues not only in Saudi Arabia but also worldwide. Investigation this issue is an urgent research area that can help to establish strategies for reducing associated harms. One approach adopted in some countries to control access to medications used to treat common minor conditions is the creation of a third class of medications that lies in the junction between prescription-only and OTC medications (3, 7), namely behind-the-counter (BTC) medications. BTC medications are dispensed under the pharmacists' professional recommendation and supervision and not simply upon patient's request.

The current global literature includes a wide range of qualitative and quantitative studies addressing OTC medication abuse and the role of pharmacists in addressing this problem (6, 8–10). In Saudi Arabia, OTC medications are widely used, with evidence suggesting that inappropriate use is linked to insufficient information offered by healthcare providers, particularly pharmacists, who represent the front line in prevention efforts (11, 12). Algarni et al. (13) conducted a semi-structured interviews with community pharmacists in the Al-Baha region, Saudi Arabia to explore their views and experiences regarding OTC medication misuse and abuse. Their qualitative study provided baseline insights into this problem and suggested potential interventions by community pharmacists. However, the overall level of awareness and preparedness among Saudi community pharmacists to control OTC-medication abuse remains unclear.

To better understand this issue, there is a need for nationwide quantitative data. Therefore, the current study was designed to be nationally representative and aimed at exploring awareness, views, and preparedness of community pharmacists to control OTC medication abuse in Saudi Arabia.

## 2 Materials and methods

### 2.1 Study design

This cross-sectional study was conducted between November 2021 and February 2022. A self-administered internet-based questionnaire with a convenient sample was used to collect data from community pharmacists across Saudi Arabia to assess their awareness of OTC medication abuse.

### 2.2 Inclusion and exclusion criteria

All registered community pharmacists of both sexes and all nationalities in Saudi Arabia were included. Pharmacy technicians, hospital pharmacists, community pharmacists not registered (unlicensed) with the Saudi Commission for Health Specialties, and those aged over 60 years were excluded.

### 2.3 Study procedure

The questionnaire was specifically designed to meet the objective of this study and was derived from relevant literature. It consisted of two parts: demographic characteristics and vigilance. The questions were written in both Arabic and English to aid clarity, reviewed by two expert academic pharmacists from the College of Pharmacy, Taif University, and modified based on the experts' feedback. The questionnaire was then evaluated for face and content validity by five pharmacy staff members from the same college. The questionnaire was piloted among 10 community pharmacists with experience in pharmacy practice and research background. The final version was then created for online completion using Google Forms, and the survey link was circulated to professional community pharmacists groups in Saudi Arabia via WhatsApp and Twitter.

### 2.4 Statistical analyses

Statistical analyses were performed using Microsoft Excel 2016 and IBM SPSS Statistics version 28.0 (IBM Corp., Armonk, NY). Descriptive statistics were presented as means (M), standard deviations (SD), frequencies (N), and percentages (%). The chi-square test and Fisher's exact test were also used to assess differences in proportions, when appropriate. The statistical significance level was set *a priori* at *p*<*0.05*.

### 2.5 Ethics and informed consent

This study was approved by the Research Ethics Committee of Taif University (reference number: HAO-02-T-105). Informed consent was obtained from all participants, who were notified that their information was entirely confidential and anonymous. Data were obtained anonymously from Google Forms and saved in an Excel spreadsheet. The identities of the participants cannot be determined.

## 3 Results

### 3.1 Demographic characteristics

A total of 813 community pharmacists completed the survey (813/919; 88.5%). Most were male (702, 86.3%), non-Saudi (623, 76.6%), bachelor's degree holders (648, 79.7%), professionally classified as pharmacists (534, 65.7%), and aged ≤ 40 years (756, 93.0%). The majority were from the western region (363, 44.6%), followed by the central (194, 23.9%), southern (148, 18.2%), and eastern regions (89, 10.9%). Most worked in chain pharmacies (808, 99.4%) or in cities (747, 91.9%). Regarding years of experience, the largest proportion had ≥10 years of experience (255, 31.4%), followed by those with ≤ 3 years of experience (217, 26.7%) and those with 7–9 years of experience (206, 25.3%) (Table 1).

**Table 1:** Demographic characteristics of the sample.

**Items**	**Measures**	**Frequency (*n* = 813)**	**Percentage (%)**
Age	≤ 30	344	42.3
	31–40	412	50.7
	41–50	52	6.4
	51–60	5	0.6
Gender	Male	702	86.3
	Female	111	13.7
Nationality	Saudi	190	23.4
	Non-Saudi	623	76.6
Educational level	B.Pharm	648	79.7
	Pharm.D	151	18.6
	Master	11	1.4
	PhD	3	0.4
Professional classification	Pharmacist	534	65.7
	Senior pharmacist	247	30.4
	Consultant pharmacist	32	3.9
Region	Western Region	363	44.6
	Central Region	194	23.9
	Southern Region	148	18.2
	Eastern Region	89	10.9
	Northern Region	19	2.3
Pharmacy location	City	747	91.9
	Village	66	8.1
Type of community pharmacy	Chain pharmacy	808	99.4
	Independent pharmacy	5	0.6
Years of experience	≥10	255	31.4
	7–9	206	25.3
	4–6	135	16.6
	≤ 3	217	26.7

B.Pharm: Bachelor of Pharmacy; Pharm.D.: Doctor of Pharmacy; Ph.D.: Doctor of Philosophy.

### 3.2 Community pharmacists' vigilance toward OTC medications abuse

Most community pharmacists were aware that some OTC medications pose a risk for addiction (570, 70.1%) and that there is a regulation or law controlling sales of OTC medications in Saudi Arabia (445, 54.7%). Using a 5-point Likert scale, pharmacists perceived their knowledge of OTC medication abuse to be high (*M* score = 4.3, SD = 0.9, 86.0%). OTC medication classes most considered to pose a risk for addiction were codeine-containing products (669, 82.2%), followed by cough suppressants/antitussives (566, 69.5%) and sleep aids (537, 66.0%). Individually, the OTC medication most considered to pose a risk for addiction was codeine (688, 84.5%), followed by dextromethorphan (534, 65.6%), and diphenhydramine (385, 47.3%). On a 5-point Likert scale, participants rated the addiction risk of individual OTC medication as follows: codeine (*M* score = 3.8, SD = 1.4, 76.0%), dextromethorphan (*M* score = 3.2, SD = 1.5, 64.0%), diphenhydramine (*M* score = 2.6, SD = 1.5, 52.0%), ephedrine (*M* score = 2.5, SD = 1.6, 50.0%), caffeine (*M* score = 2.2, SD = 1.6, 44.0%), and pseudoephedrine (*M* score = 2.2, SD = 1.6, 44.0%) (Table 2).

**Table 2:** Community pharmacists' vigilance towards OTC medications abuse.

**Variable**		**Frequency (n = 813)**	**Percentage (%)**
**According to your knowledge, is there a regulation or law**			
**controlling sales of OTC medications in Saudi Arabia?**			
	Yes	445	54.7
	No	169	20.9
	Not sure	199	24.4
**According to your knowledge, do some OTC medications pose**			
**a risk for developing an addiction?**			
	Yes	570	70.1
	No	163	20.0
	Not sure	80	9.8
**According to your knowledge, do the following OTC classes**			
**pose a risk for developing an addiction?**			
Codeine-containing products	Yes	669	82.2
	No	67	8.2
	Not sure	77	9.5
Cough suppressants/Antitussives	Yes	566	69.5
	No	160	19.7
	Not sure	87	10.7
Sleep aids	Yes	537	66.0
	No	176	21.6
	Not sure	100	12.3
Decongestants	Yes	318	39.1
	No	380	46.7
	Not sure	115	14.1
Cold medicines	Yes	236	29.0
	No	437	53.7
	Not sure	140	17.2
Expectorants	Yes	119	14.6
	No	547	67.2
	Not sure	147	18.1
**According to your knowledge, do the following OTC**			
**medications pose a risk for developing an addiction?**			
Codeine	Yes	688	84.5
	No	63	7.7
	Not sure	62	7.6
Dextromethorphan	Yes	534	65.6
	No	188	23.1
	Not sure	91	11.2
Diphenhydramine	Yes	385	47.3
	No	304	37.3
	Not sure	124	15.2
Ephedrine	Yes	329	40.4
	No	340	41.8
	Not sure	144	17.7
Caffeine	Yes	254	31.2
	No	442	54.3
	Not sure	117	14.4
Pseudoephedrine	Yes	242	29.7
	No	450	55.3
	Not sure	121	14.9
**Are you familiar with the concept of BTC medications?**			
	Yes	738	90.8
	No	21	2.6
	Not sure	54	6.6
**Do you think transition of OTC medications with abuse**			
**potential to a BTC status will help reducing OTC medication**			
**abuse?**			
	Yes	527	64.8
	No	108	13.3
	Not sure	178	21.9
**Normally, how do you deal with a suspicious patient/customer**			
**who may be abusing an OTC medication?**			
Provide advice to patients as necessary	Yes	707	87.0
	No	106	13.0
Refer to a physician	Yes	696	85.6
	No	117	14.4
Provide information leaflets and counseling about the abuse potential of the product	Yes	597	73.4
	No	216	26.6
Refuse sales	Yes	562	69.1
	No	251	30.9
Investigate the patient/customer suspicious behavior	Yes	540	66.4
	No	273	33.6
Contact other pharmacies to warn them of the suspicions of a customer who may be abusing a product	Yes	521	64.1
	No	292	35.9
Claim products were not in stock	Yes	466	57.3
	No	347	42.7
Do nothing and providing the requested products	Yes	219	26.9
	No	594	73.1
**How do you perceive your knowledge about OTC medications**			
**abuse? (0 not at all - 5 excellent)**			
	4.3 (0.9)		86.0
**If some OTC medications pose a risk for developing an**			
**addiction, to what extent such risk is high? (0 no risk - 5**			
**highest risk)**			
	3.0 (1.3)		60.0
**If the following OTC medications pose a risk for developing an**			
**addiction, to what extent such risk is high? (0 no risk - 5**			
**highest risk)**			
Codeine	3.8 (1.4)		76.0
Dextromethorphan	3.2 (1.5)		64.0
Diphenhydramine	2.6 (1.5)		52.0
Ephedrine	2.5 (1.6)		50.0
Caffeine	2.2 (1.6)		44.0
Pseudoephedrine	2.2 (1.6)		44.0
**According to your opinion, to what extent the following**			
**represent important barriers for pharmacists to prevent OTC**			
**medications abuse? (0 least important - 5 most important)**			
Lack of patient records on OTC medications uses	3.2 (1.4)		64.0
Workload	3.1 (1.5)		62.0
Lack of consistent data on OTC medications	2.8 (1.5)		56.0
Shortage of workforce in a pharmacy led to reduced focus/attention	2.8 (1.5)		56.0
Lack of information about OTC drug related problems	2.7 (1.5)		54.0

OTC, Over-The-Counter; BTC, Behind-The-Counter.

Dealing with a suspicious patient/customer who may be abusing OTC medication, most respondents reported that they would provide advice to patients as necessary (707, 87.0%), refer them to a physician (696, 85.6%), provide information leaflets and counseling about the abuse potential of the product (597, 73.4%), refuse sale (562, 69.1%), and investigate suspicious patient/customer behavior (540, 66.4%). On a 5-points Likert scale, the top-rated barriers for preventing OTC medication abuse included lack of patient records on OTC medication use (*M* score = 3.2 5, SD = 1.4, 64.0%), workload (*M* score = 3.1, SD = 1.5, 62.0%), lack of consistent data on OTC medications (*M* score = 2.8, SD = 1.5, 56.0%), and shortage of workforce in a pharmacy, leading to reduced focus/attention (M score = 2.8, SD = 1.5, 56.0%). Most respondents were familiar with the concept of BTC medications (738, 90.8%) and believed that the transition of OTC medications with abuse potential into the BTC category could help reduce OTC medication abuse (527, 64.8%) (Table 2).

### 3.3 Community pharmacists' vigilance toward OTC medications abuse by respondents characteristics

Statistically significant differences were observed in responses to the question, “How do you perceive your knowledge about OTC medication abuse?” by years of experience and professional classification (*P* < 0.05 for both; Table 3). Those with greater years of experience (i.e., 7–10) had higher perceptions of their knowledge about OTC medication abuse. Similarly, consultants had the highest perceived knowledge of OTC medication abuse, followed by senior pharmacists.

**Table 3:** Community pharmacists' vigilance toward OTC medications abuse by respondents characteristics.

**Items**	**Comparisons**				
**How do you perceive your knowledge about OTC medications abuse?**	**Years of experience**				**P-Value**
	≤ **3**	**4-6**	**7-9**	≥**10**	
0	4 (1.8%)	2 (1.5%)	0 (0.0%)	0 (0.0%)	
1	0 (0.0%)	0 (0.0%)	1 (0.5%)	1 (0.4%)	
2	10 (4.6%)	1 (0.7%)	3 (1.5%)	3 (1.2%)	
3	51 (23.5%)	13 (9.6%)	11 (5.3%)	21 (8.2%)	< 0.05
4	75 (34.6%)	51 (37.8%)	75 (36.4%)	95 (37.3%)	
5	77 (35.5%)	68 (50.4%)	116 (56.3%)	135 (52.9%)	
Total	217 (100.0%)	135 (100.0%)	206 (100.0%)	255 (100.0%)	
**According to your knowledge, do some OTC medications pose a risk for developing an addiction?**	**Years of experience**				**P-Value**
	≤ **3**	**4-6**	**7-9**	≥**10**	
Yes	174 (80.2%)	91 (67.4%)	129 (62.6%)	176 (69.0%)	
No	27 (12.4%)	34 (25.2%)	60 (29.1%)	42 (16.5%)	< 0.05
Not sure	16 (7.4%)	10 (7.4%)	17 (8.3%)	37 (14.5%)	
Total	217 (100.0%)	135 (100.0%)	206 (100.0%)	255 (100.0%)	
**How do you perceive your knowledge about OTC medication abuse?**	**Professional classification**			
	**Pharmacist**	**Senior pharmacist**	**Consultant pharmacist**	**P-Value**
0	6 (1.1%)	0 (0.0%)	0 (0.0%)	
1	2 (0.4%)	0 (0.0%)	0 (0.0%)	
2	14 (2.6%)	3 (1.2%)	0 (0.0%)	
3	84 (15.7%)	11 (4.5%)	1 (3.1%)	< 0.05
4	190 (35.6%)	97 (39.3%)	9 (28.1%)	
5	238 (44.6%)	136 (55.1%)	22 (68.8%)	
Total	534 (100.0%)	247 (100.0%)	32 (100.0%)	
**According to your knowledge, do some OTC medications pose a risk for developing an addiction?**	**Region**					**P-Value**
	**Western**	**Central**	**Southern**	**Eastern**	**Northern**	
Yes	276 (76.0%)	116 (59.8%)	96 (64.9%)	68 (76.4%)	14 (73.7%)	
No	61 (16.8%)	52 (26.8%)	33 (22.3%)	14 (15.7%)	3 (15.8%)	
Not sure	26 (7.2%)	26 (13.4%)	19 (12.8%)	7 (7.9%)	2 (10.5%)	< 0.05
Total	363 (100.0%)	194 (100.0%)	148 (100.0%)	89 (100.0%)	19 (100.0%)	

OTC, Over-The-Counter.

Significant differences were also observed in responses to the question, “do some OTC medications pose a risk for developing an addiction?” by years of experience and work region (*P* < 0.05 for both). Those with fewer years of experience had higher perceptions of OTC medication risk for developing an addiction. Participants from the western, eastern, and northern regions reported higher perceptions of OTC medication risk for developing addiction compared with their counterparts.

## 4 Discussion

To our knowledge, this is the first nationwide quantitative study in Saudi Arabia on community pharmacists' preparedness to control OTC medication abuse. Previous research has mainly consisted of qualitative studies on pharmacists' views on OTC medication abuse in the country (13) or those addressing medication abuse in general without specifically focusing on OTC medication abuse (14). Other studies targeted populations other than pharmacists (11). These features are reflective of most studies conducted in the Gulf Region and worldwide (15, 16). Moreover, previous studies have generally involved smaller sample sizes compared with the current study. Therefore, the present study represents a valuable source of information on this topic, with a particular focus on the preparedness of community pharmacists to address OTC medication abuse.

This study revealed adequate preparedness among the participating community pharmacists. They perceived their knowledge of OTC medication abuse to be high and rated the risk of abuse across various medications, with codeine receiving the highest rating (84.5%) and pseudoephedrine the lowest (29.7%). Pharmacists determined how they typically deal with customers suspected of OTC medication abuse and identified important barriers that hinder them from preventing such abuse. Therefore, pharmacists should be knowledgeable about OTC medication abuse. In addition to their basic qualifications and formal training during undergraduate studies, some pharmacists receive on-the-job training as a means of continuing the professional education required for license renewal.

Previous literature from Saudi Arabia indicated that most community pharmacists received training on drug misuse and abuse and showed interest in receiving further training. The majority also provided advice and education to their patients about drug abuse and misuse (14). In our sample, 87% reported providing advice to patients when necessary, and 73.4% provided information leaflets and counseling about the abuse potential of the abuseable product. A qualitative study from this country revealed that community pharmacists identified counseling customers on the appropriate use of medicines, providing safe alternatives, and refusing to sell products as common strategies used to address misuse or abuse (13). In the present study, a substantial proportion of pharmacists (69.1%) reported refusing sales (69.1%) and claiming products were out of stock (57.3%) as possible ways to deal with a suspicious customer who may be abusing OTC medications.

Community pharmacists' efforts to control OTC medication abuse are challenging. Wazaify et al. (16) applied and tested an intervention model that minimized OTC drug misuse and abuse in community pharmacy. Pharmacists enrolled in that study reported difficulties in approaching potential customers suspected of abuse.

In the present study, most pharmacists identified codeine as posing the highest risk of addiction (84.5%), followed by dextromethorphan (65.6%), diphenhydramine (47.3%), ephedrine (40.4%), caffeine (31.2%), and pseudoephedrine (29.7%). Although it was expected that more pharmacists would rate these medications as potentially abusable substances with addictive properties, their ratings reflected the degree of risk seriousness of such medications. Among children and teenagers, Crouch et al. (17) found that the most commonly abused non-prescription medications were those with anticholinergic properties, caffeine, dextromethorphan, and non-prescription stimulants. Thekiso and Farren (18) examined the clinical profiles, treatments, and prevalence of opiate abuse among patients admitted for OTC drug misuse. They reported that 75% of inpatients admitted and diagnosed with OTC opioid abuse experienced withdrawal symptoms and were treated with protocol-driven withdrawal regimens for an average of 16.10 days. The authors concluded that “OTC opiate abuse is a significant problem which is largely covert in nature, with serious co-morbidity and frequent complications including, withdrawal symptoms which require treatment.” This finding reflects the seriousness of the abuse of such medications and other OTC abusable drugs. In the same context, Ahmed described a report of 33 men in Pakistan who lost their lives due to an overdose of cough syrup containing dextromethorphan (19). The authors stated, “Abuse of illicit drugs such as cough medicines, which are available OTC, is an area often overlooked and usually under stressed.”

A strength of the current study was its relatively large sample size and being representative of different regions of Saudi Arabia specifically with a good representation of the most populous areas of the country like the western and central parts. However, a limitation of the study was the online nature of the survey and the convenient sampling technique which are prone to selection bias and information bias more than face to face survey and probability sampling. However, both are widely used in research and are acceptable by the scientific community.

In conclusion, most community pharmacists in Saudi Arabia are aware of OTC medication abuse. Establishment of clear policies and guidelines regarding purchasing limits and monitoring of OTC abuse is urgently needed. Future studies should be aimed at determining the impact of newly established drug policies, guidelines, and/or monitoring programs targeting OTC medications.

## Data Availability

The raw data supporting the conclusions of this article will be made available by the authors, without undue reservation.
